# Relationship between Medication Use and Cardiovascular Disease Health Outcomes in the Jackson Heart Study

**DOI:** 10.3390/ijerph8062505

**Published:** 2011-06-23

**Authors:** Clifton C. Addison, Brenda W. Jenkins, Daniel Sarpong, Gregory Wilson, Cora Champion, Jeraline Sims, Monique S. White

**Affiliations:** 1 Jackson Heart Study/Project Health, Jackson State University, 350 W. Woodrow Wilson Drive, Suite 701, Jackson, MS 39213, USA; E-Mails: brenda.w.campbell@jsums.edu (B.W.J.); mswhite427@yahoo.com (M.S.W.); 2 Jackson Heart Study, Jackson State University, 350 W. Woodrow Wilson Drive, Suite 701, Jackson, MS 39213, USA; E-Mails: dannys107@yahoo.com (D.S.); gregory.wilson@jsums.edu (G.W.); 3 Jackson Heart Study, Department of Medicine, University of Mississippi Medical Center, 350 W. Woodrow Wilson Drive, Suite 701, Jackson, MS 39213, USA; E-Mails: cchampion@umc.edu (C.C.); jsims@umc.edu (J.S.)

**Keywords:** medication use, minority, African-Americans, Jackson Heart Study, cardiovascular disease

## Abstract

Even though some medications have the potential to slow the progress of atherosclerosis and development of CVD, there are many at-risk individuals who continue to resist the benefits that are available by not following the advice of medical professionals. Non-adherence to prescribed drug regimens is a pervasive medical problem that negatively affects treatment outcomes. Information from standardized interviews of 5301 African Americans participating in the Jackson Heart Study was examined to determine the association between demographic parameters, behavior including adherence to prescribed medical regimens, and health outcomes. Data were also collected at Annual Follow-Up and Surveillance visits. During the two weeks prior to the examination visit, almost 52% of the participants reported taking blood pressure medication, 14% took cholesterol medication, 16% took medication for diabetes, and 19% took blood thinning medication. Of those who did not take the prescribed medications, the reasons given were the following: 47% were in a hurry, too busy, or forgot to take medications; 23% were trying to do without medications; 18% had no money to purchase medications; 19% indicated that the medications made them feel bad; 17% felt that they could not carry out daily functions when taking medications. The African American population can benefit from heightened awareness of the risk factors that are associated with CVD and the benefits of following a prescribed treatment regimen. Unacceptable secondary effects of prescribed medication comprised an important cause of non-compliance. Encouragement of this population to communicate with their healthcare providers to ensure that medication regimens are better tolerated could increase compliance and improve health outcomes.

## 1. Introduction

In recent years, medical science has reported some degree of progress in the struggle against cardiovascular disease (CVD), but it remains a major health concern, accounting for approximately 1 million deaths in the United States each year [1]. Progress has been attributed to strategic active prevention of the various risk factors, improvements in healthcare, and the development and use of new therapies. Even though the potential of some medications to slow the progress of atherosclerosis and the development of CVD has received wide exposure, large numbers of individuals resist medication adherence and fail to take advantage of the potential benefits of a daily medical regimen [2]. Medication non-adherence reduces the effectiveness of therapies.

Munger, Van Tassell, & LaFleur [3] have referred to non-adherence as an unrecognized risk factor and as a pervasive medical problem that negatively affects treatment outcomes. One third to one half of patients do not comply with prescribed treatment regimens [4–6]. Non-adherent patients have also exhibited higher glycosylated hemoglobin, systolic and diastolic blood pressure, and low-density lipoprotein cholesterol levels [7]. Non-adherence has been identified as a frequent cause of relapse among patients [8]. Immune suppression therapy non-adherence is the leading avoidable cause of renal transplant failure. Renal transplant patients particularly at risk may be those who have a history of non-adherence to medical advice [9]. Between one third and two thirds of all medication-related hospital admissions are attributed to non-adherence [10], costing the healthcare system an estimated $ 792 million to $1 billion annually [4,11].

In patients with hypertension, medication non-adherence is a significant, often unrecognized, risk factor that contributes to poor blood pressure control, which could later lead to the development of more serious disorders like heart failure, coronary heart disease, renal insufficiency, and stroke [3]. One study found that fewer than 50% of patients who were prescribed statin medications were adherent 12 months after initiating treatment, and other research has demonstrated that non-adherence to oral hypoglycemics and antihypertensive medications has been associated with higher HbA_1c_ and BP levels, respectively [12,13].

The practice of non-adherence results in only 59% of people with hypertension regularly receiving effective treatment [14]. Hypertension increases the chances for stroke, end-stage renal disease, and heart failure, as well as contributes to the prevalence of insulin resistance, lipid abnormalities, changes in renal function, endocrine abnormalities, obesity, left ventricular hypertrophy, diastolic dysfunction, and abnormalities in vascular structure and elasticity [15]. Even though effective antihypertensive drugs are available, poor adherence to prescribed treatment remains a major cause of uncontrolled blood pressure that could result in target organ damage and increased cardiovascular risk. Medication adherence rates have been classified in ranges from 35% to 97% [16,17].

Older adults are prescribed more medication than any other group, and poor adherence is a common reason for their non-response to medication [18]. More needs to be done to promote adherence, heighten awareness, and understand the reasons for the resistance to prescribed treatment regimen in the African American community with its high prevalence of CVD [19]. Social support has been shown to have both direct and indirect associations with medical adherence [20]. It is important for individuals to understand that those who have had a heart attack previously are at an increased risk of death when they do not adhere to the prescribed medication [6], especially since about one third to two thirds of all patients admitted to the hospital with medication-related problems are those who have not been following the prescribed medication program [10,21]. In one study, about half of the patients who were prescribed an antihypertensive drug had stopped taking it within one year. The likelihood that a patient would discontinue treatment early was inversely related to the quality of regular execution of the dosing regimen [22]. Therapeutic approaches that are designed to improve treatment adherence attitudes and behaviors should address an individual’s risk practices and the potential negative health outcomes [8].

Few studies have comprehensively examined adherence to medication regimen in African-Americans and possible health outcomes associated with medication adherence when diagnosed with disease. One study reported that even though African Americans tend to have higher rates of diabetes and disease-related complications, they had a 12% lower medication adherence rate than whites. These low rates of adherence have been linked to lower socioeconomic status and to lower levels of education. African-Americans with type 2 diabetes appear to be less likely than whites to take prescribed medications [23]. The Jackson Heart Study has collected data on a large cohort of African-Americans and provides an appropriate mechanism to explore medication adherence in this group.

The aim of this study was to assess CVD diagnoses and adherence to CVD medications, and to identify predictors of hospitalization for CVD conditions. It was hypothesized that JHS participants who adhered to a prescribed medical regimen for a diagnosed CVD condition would encounter less deterioration in CVD health status (as measured by hospitalization) than those who did not adhere to a prescribed medical regimen for a diagnosed CVD condition. The study examined the following research questions:

1. What is the extent of cardiovascular disease diagnoses in the Jackson Heart Study cohort?
2. What is the extent of medication adherence in the Jackson Heart Study cohort?
3. What are the predictors of cardiovascular disease hospitalization among JHS participants?
4. What are the reasons for non-adherence in the JHS?

## 2. Experimental Section

### 2.1. Methods

Baseline interview data collected from 5301 African Americans, living in the tri-county area of metropolitan Jackson, Mississippi; Hinds, Madison and Rankin counties and participating in the Jackson Heart Study, were analyzed. The Jackson Heart Study (JHS) is the largest single-site epidemiological study of cardiovascular disease on African-Americans on record. A systematic review of the JHS data was made focusing on medication use, CVD health status, and demographic parameters to examine practices and behaviors of the participants and association between those practices and the primary health outcome, hospitalization. Details of the JHS, its development, participants, and overall procedures are described further by Addison *et al.* [24] and Campbell-Jenkins *et al*. [25] in earlier publications.

Demographic variables of the JHS participants (age, education level, age, income, and county of residence) were extracted from the Household Enumeration Form (HEF) and Personal Data— Socioeconomic Status Form (PDS). Cardiovascular conditions of JHS participants at baseline (high blood pressure, high cholesterol, heart attack, diabetes, and stroke) were obtained from the Personal and Family History Form (PFHA). On this form, participants responded with a “yes”, “no”, or “don’t know” answer about whether they were told by a physician that they had certain diseases. JHS participants’ CVD medication use was obtained from the Medication Survey Form (MSRA). On this form, the participants were again asked to respond with a “with a “yes”, “no”, or “don’t know” answer about whether they had taken medication for high blood pressure, high cholesterol, heart attack, diabetes, or stroke during the two weeks prior to coming to the Exam Center for their clinic exams. Self-report of adherence to medications is a useful and expedient way of assessing adherence [26]. JHS participants’ reasons for not taking the doctor-prescribed medication were also elicited from the MSRA. Participants were given a list of potential reasons cited that prevented people from taking medication as prescribed, and asked to indicate with a “yes”, “no”, or “don’t know” answer. The primary study outcome was self-reported hospitalization for CVD which was extracted from the participants’ annual follow-up interviews. Subjects were asked at 1-year follow-up interviews whether they had been hospitalized for CVD during the previous 12 months.

### 2.2. Statistical Analysis

We calculated CVD health status among participants by age, gender, education, and income. We ascertained adherence to medication among participants with self-reported CVD-related morbidity by age, gender, education, and income. Differences in the occurrence of hospitalizations between participants who adhere to a medical regimen and those who do not were assessed. Because medication non-adherence may act on the causal pathway between a risk factor and subsequent hospitalization for CVD, this study evaluated medication adherence as a risk factor for CVD-related hospitalization, taking baseline CVD conditions into account. We analyzed the binary outcome of hospitalization or no-hospitalization for CVD-related health status using logistic regression analysis, with separate models for each predictor variable to assess the association between the medication adherence of diagnosed participants. All statistical tests were considered significant at the 0.05 level. The SAS version 9.2 (SAS Institute, Cary, North Carolina) and SPSS 18.0 were used for all statistical analyses, which were performed independently at Jackson Heart Study Coordinating Center (Jackson, Mississippi).

## 3. Results and Discussion

### 3.1. Results

In Table 1, the distribution of self-reported cardiovascular conditions of the JHS participants at baseline are presented. The JHS participants provided responses to the questions regarding whether they were told by a doctor that they had high blood pressure, high cholesterol, heart attack, diabetes, kidney problems, or stroke. The highest number of high blood pressure diagnoses occurred with the older participants aged 65 and older. However, it is noteworthy that over 14.0% of the youngest age group 21–34 indicated that they were diagnosed with high blood pressure. High cholesterol diagnoses seem to occur more frequently in the over 65 year age group, with almost 4.0% of the youngest group, 21–34, also classified in this category. The rate of diabetes diagnoses was higher than the national average in the JHS participants over the age of 45. JHS participants aged 21–34 (4.8%) and 35–44 (7.3%) had a significant number who were diagnosed with diabetes. The largest group of diagnoses with kidney problems was the over 85 group (26.7%). However, the 21–34 age group (8.4%) surfaced as having a larger number of kidney problem diagnoses than participants 35–74. Only the 75–84 age group with 9.9% of the cases was higher than the 21–34 age group, with the exception of the over 85 age category with its 26.7%. The diagnosis of stroke appeared to occur with more frequency in the older age groups, with the over 85 group having the greatest representation (20.0%).

A comparison of the diagnoses made by a doctor based on gender showed that females were more likely than males to have diagnoses in four of the six categories examined. Females were also more likely than males to have high blood pressure (58.3% to 49.7%), high cholesterol (28.8% to 25.6%), diabetes (18.4% to 15.3%), and kidney problems (5.6% to 4.3%). The male JHS participants had more diagnoses in the area of heart attacks (6.4% to 4.1%) and strokes (4.2% to 3.4%).

We examined the diagnoses made by a doctor based on education level. Greater educational attainment seemed to translate into a lower degree of CVD related diagnoses. In every category of diagnoses, the JHS participants with the lowest level of education (< high school) experienced the greatest degree of medical issues, leading the way with blood pressure diagnoses of 70.8% and diabetes diagnoses of 26.2%. This group also had a stroke diagnosis rate of 8.4% compared to 1.9% for the participants who had a Bachelors degree and above and a heart attack rate of 9.3% compared to the 2.9% for the group with a Bachelors degree or above. In all of these disease categories, the data reveal that the amount of CVD related diagnoses occurs more frequently the lower one appears on the education level spectrum. Income level seems to have played a part in the distribution of the CVD-related diagnoses as reported by the JHS participants. In all of the disease categories, JHS participants in the lowest income levels reported a larger number of diagnoses of high blood pressure, cholesterol, heart problems, diabetes, kidney problems, and strokes. High cholesterol is the only illness category where all of the JHS participants seem to be affected more evenly.

Table 2 examines the medication adherence and non-adherence of the JHS African-American cohort based on the diagnoses of these conditions reported to them by a doctor. Of the five categories examined in this table, two categories, high blood pressure and diabetes, had high adherence rates of 86.6% and 88.3% respectively. On the other hand, adherence rates for those JHS participants diagnosed with high cholesterol, heart problems, and strokes were exceptionally low. Only 13.0% of the African-American participants in the JHS adhered to the recommended heart medications. Equally of concern was the adherence rate for JHS participants who were diagnosed with a stroke which was estimated at 22.2%. JHS participants who were diagnosed with high cholesterol also demonstrated poor adherence rates with a rate of 39.7%.

Regression results indicated the overall fit of the predictors was moderate (−2 Log Likelihood = 1591.993). The model was statistically reliable in distinguishing between hospitalization of JHS participants *X*^2^ (6) = 45.050, p < 0.05. The model correctly classified 88.6% of the cases. Regression coefficients are presented in Table 3. Wald statistics indicated that the chance of being hospitalized for a CVD- related illness was increased for JHS participants who were female and those participants who did not adhere to their medication for treating chest pains and diabetes.

Table 4 provides a ranking of the top ten reasons cited by the JHS participants for not taking their medications. The number one reason cited for creating this lapse in practice was that participants got in too much of a hurry and forgot to take their medications. Almost half of the participants (47.3%) recognized the busy lifestyle as a deterrent to maintaining a regular medical regimen. About one quarter of the participants (23.5%) believed that they could combat their medical issues without the aid of the prescriptions, and over 20.0% of the participants did not take their medications because the medications were not available to them. Approximately 19.0% participants of the JHS admitted that they did not take their medications because the medications made them feel bad. Over 18.0% of the JHS participants indicated that the high cost of living and the cost of medication made it difficult for them to acquire their prescriptions, and about 17.0% of them viewed the medication regimen as an obstacle to their normal daily activities. The discomforts they experienced prevented them from engaging in effective medical adherence. More than 13.0% of the participants believed that the prescribed medication created an inconvenience that they could not overcome and this caused them to become delinquent in their treatment regimen. About 10.0% of the participants had little faith in the efficacy of the medical regimen and desisted adherence, and 10.0% of the participants feared that they might become addicted to the medication so they refused to fill their prescriptions. In addition, 10.0% of the participants simply did not like taking medication, so they abstained from practicing adherence.

## 4. Conclusions

A high adherence rate was found in this sample of 5301 for diabetes and high blood pressure medication in spite of the fact that African-Americans are less likely than whites to take prescribed medications, and African Americans with type 2 diabetes tend to have comparatively poor clinical outcomes. Even though the adherence rate for diabetes and hypertension medication was high, it is evident that there are still a considerable number of African-Americans who do not adhere to their daily medical regimen. The research also demonstrated that there are many African-Americans who do not trust the utility of the medications prescribed by their doctor. Observed disparities in outcomes for African-Americans could be attributed in part to the low acceptance of evidence-based therapies, resulting in some degree of medication non adherence. It is evident that some participants do not accept the suggestion that daily compliance could reduce the need for subsequent medication and treatment to reduce the risk for complications.

Belonging to the female gender and non-adherence of heart and stroke medication have been associated with a greater risk of hospitalization for CVD. In this study, these variables were seen as being most strongly predictive of subsequent hospitalization. The percentage of participants not adherent to medication after being diagnosed with high cholesterol, heart failure, and stroke is high. A possible result of non-adherence is the worsening of medical conditions and other negative health outcomes. A majority of the Jackson Heart Study participants reported that they did not take their medications after having been diagnosed with stroke, cholesterol problems, and heart attacks. Inadequate preventive CVD care has been frequently cited as a contributing factor to negative health outcomes.

These results can guide the initiation of quality improvement interventions to target African-American CVD patients who might be in need of strong self-management guidance and support. Medication adherence should be a key component of any disease self-management plan that intends to present intervention strategies to effectively support constructive preventive care. It is imperative that culturally sensitive education material be developed and implemented to effectively manage CVD, and we propose the formation of support groups for promoting adherence to treatment among African Americans. The community should be exposed to information regarding eliminating the factors contributing to non-adherence to medical regimens for CVD as they relate to the reduction in health disparities and the reduction in morbidity, hospitalization and mortality among this African-American population. Our results provide evidence that adherence to prescribed medication is influenced by a multitude of factors, and we advocate strong support for interventions to increase medication adherence and decrease adverse outcomes so that patients can realize the full benefit of prescribed therapies and participate in improving the quality of their lives. Public health officials and medical practitioners must first identify those individuals who are at greatest risk for non-adherence and then develop a proactive management program that highlights daily medical compliance and persistence with the prescribed drug regimen.

### 

#### Limitations

The main limitation of this study is the reliance on self-reported medication use and CVD conditions data. Participant differences in recall or reporting of medication use, health conditions, and hospitalization could influence the risk model.

## Figures and Tables

**Table 1.** Self-reported cardiovascular conditions of JHS participants at baseline.

**Characteristics**			**Told by Doctor Had High Blood Pressure**		**Told by Doctor Had High Cholesterol**		**Told by Doctor Had Heart Attack**		**Told by Doctor Had Diabetes**		**Told by Doctor Had Kidney Problems**		**Told by Doctor Had Stroke**	
			p < 0.05		p > 0.05		p > 0.05		p > 0.05		p> 0.05		p > 0.05	
**Age**	N	%	N	%	N	%	N	%	N	%	N	%	N	%
21–34	251	4.7	36	**14.3**	10	**3.9**	0	**0.0**	12	**4. 8**	21	**8.4**	1	**0.4**
35–44	1008	19.0	340	**33.7**	159	**15.8**	9	**0.9**	74	**7.3**	31	**3.1**	7	**0.7**
45–54	1302	24. 6	620	**47.7**	338	**26.0**	50	**3.9**	176	**13.6**	54	**4.2**	19	**1.5**
55–64	1436	27.1	949	**56.2**	463	**32.3**	88	**6.1**	318	**22.2**	72	**5.0**	72	**5.0**
65–74	996	18.8	741	**74.6**	383	**38.6**	85	**8.6**	256	**25.8**	60	**6.0**	75	**7.6**
75–84	283	5.5	226	**77.0**	102	**34.9**	29	**9.9**	75	**25.7**	29	**9.9**	18	**6.2**
Over 85	15	0.3	10	**66.7**	5	**33.3**	1	**6.7**	1	**6.7**	4	**26.7**	3	**20.0**
**Sex**			p > 0.05		p > 0.05		p > 0.05		p > 0.05		p > 0.05		p > 0.05	
Female	3360	63.4	1958	**58.3**	965	**28.8**	138	**4.1**	616	**18.4**	188	**5.6**	113	**3.4**
Male	1941	36.6	964	**49.7**	495	**25.6**	124	**6.4**	296	**15.3**	83	**4.3**	82	**4.2**
**Education Level**			p > 0.05		p < 0.05		p < 0.05		p > 0.05		p > 0.05		p < 0.05	
Less than High School	973	18.42	690	**70.8**	329	**32.9**	90	**9.3**	255	**26.2**	81	**8.3**	82	**8.4**
High School/GED	1064	20.15	630	**59.2**	289	**27.2**	64	**6.0**	183	**17.2**	63	**5.9**	39	**3.7**
>High School,< Bachelors	1525	28.88	749	**49.1**	376	**24.7**	56	**3.7**	232	**15.2**	63	**5.9**	41	**2.7**
Bachelors degree or higher	1719	32.55	843	**49.1**	462	**26.9**	51	**2.9**	239	**13.9**	62	**3.6**	33	**1.9**
**Family Income**			p > 0.05		p > 0.05		p > 0.05		p > 0.05		p > 0.05		p > 0.05	
Low	701	15.64	432	**61.6**	201	**28.7**	57	**8.1**	160	**22.8**	64	**9.1**	52	**7.4**
Lower–Middle	1131	25.24	691	**61.1**	328	**29.0**	65	**5.8**	219	**19.3**	61	**5.4**	60	**6.3**
Upper–Middle	1328	29.64	690	**52.0**	338	**25.5**	49	**3.7**	208	**15.7**	44	**3.3**	37	**2.8**
Affluent	1321	29.48	650	**49.2**	354	**26.8**	37	**2.8**	164	**12.4**	38	**2.9**	18	**1.4**
**County of Residence**			p > 0.05		p > 0.05		p > 0.05		p > 0.05		p > 0.05		p > 0.05	
Hinds	4367	82.44	2472	**56.7**	1200	**27.5**		**5.0**	768	**17.6**	232	**5.3**	170	**3.9**
Madison	498	9.40	244	**49.0**	158	**31.7**	23	**4.6**	80	**16.1**	27	**5.2**	13	**2.6**
Rankin	232	4.38	107	**46.3**	54	**23.4**	8	**3.5**	28	**12.1**	7	**3.0**	5	**2.2**
Unknown	200	3.78	97	**48.5**	48	**24.0**	15	**7.5**	35	**17.5**	4	**2.0**	7	**3.5**

**Table 2.** Medication adherence by cardiovascular conditions of JHS participants at baseline.

**Cardiovascular Condition**	Took High Blood Pressure Medication	Took Cholesterol Medication	Took Heart Failure Medication	Took Diabetes Medication	Took Stroke Medication
Told by Doctor had High Blood Pressure **N = 2845**	**Yes = 2464 (86.6)** **No = 360 (13.4)**				
Told by Doctor had High Cholesterol **N = 1405**		**Yes = 558 (39.7)** **No = 844 (60.1)**			
Told by Doctor had Heart Attack **N = 254**			**Yes = 33 (13.0)** **No = 220 (86.6)**		
Told by Doctor had Diabetes **N = 888**				**Yes = 784 (88.3)** **No = 104 (11.7)**	
Told by Doctor had Stroke **N = 189**					**Yes = 42 (22.2)** **No = 147 (77.8)**

**Table 3.** Factors related to JHS hospitalization-regression coefficients.

	B	S.E.	Wald	df	Sig.	Exp (B)

Female	0.307	0.148	4.290	1	0.038	1.359
Past 2 weeks, did not take medication for angina/chest pains	−1.179	0.240	24.158	1	0.000	0.308
Past 2 weeks, did not take medication for diabetes/high blood sugar	−0.336	0.148	5.191	1	0.023	0.714
Constant	−0.915	0.296	10.373	1	0.001	0.385

**Table 4.** JHS participants’ top ten reasons for not taking medications.

Top Ten Ranked Reasons	%
1. In a hurry, too busy, forgot to take medications.	47.3
2. Trying to do without taking medications.	23.5
3. Did not have medication available.	21.1
4. Medication made you feel bad-did not take.	18.9
5. No money to purchase medication.	18.2
6. Can’t carry out normal activities-did not take medications.	17.1
7. Medication was inconvenient to take.	13.3
8. Medication wouldn’t do any good-did not take.	10.5
9. Thought you might become addicted-did not take medications.	10.3
10. Don’t like to take medicine.	9.9
